# Trypanorhyncha (Cestoda) from Endemic *Rhinobatos* Species (Elasmobranchii: Rhinobatidae) from Bali, Indonesia, with the Description of Two New Species

**DOI:** 10.1007/s11686-026-01365-3

**Published:** 2026-09-26

**Authors:** Xaver Neitemeier-Duventester, Endang Wulandari Suryaningtyas, Jorina Wiebke Riegert, I Made Damriyasa, Wayan Arthana, Harry Wilhelm Palm

**Affiliations:** 1 Aquaculture and Sea‑Ranching, Faculty of Agriculture, Civil and Environmental Engineering, University of Rostock, Justus‑Von‑Liebig‑Weg 6, 18059 Rostock, Germany; 2 Aquaculture Study Program, Faculty of Marine Science and Fisheries, Udayana University, Kampus Bukit Jimbaran, 80361 Kuta Selatan, Bali, Indonesia; 3 Aquatic Resources Management Study Program, Faculty of Marine Science and Fisheries, Udayana University, Kampus Bukit Jimbaran, Kuta Selatan, Bali, 80361 Indonesia; 4 Faculty of Veterinary Medicine, Udayana University, Kampus Bukit Jimbaran, Kuta Selatan, Bali, 80361 Indonesia

**Keywords:** *Prochristianella bicki* sp. nov., *Pseudochristianella jimbaranensis* sp. nov., Taxonomy, Confocal laser scanning microscopy, Life cycle, Feeding ecology

## Abstract

**Purpose:**

The aim of this study was to analyze the Trypanoryncha fauna of *Rhinobatos jimbaranensis* Last, White & Fahmi, 2006 and *R. penggali* Last, White & Fahmi, 2006 from Bali, Indonesia, with respect to their presumed feeding ecology.

**Methods:**

Twenty-three ray specimens of the family Rhinobatidae were purchased at the Pasar Ikan Tradisional Kedonganan market on the southern coast of Bali, Indonesia, and examined for the trypanorhynchs using standardized methods. Between 2018 and 2021, 12 *R. jimbaranensis* and 11 *R. penggali* were examined, both species are endemic to Indonesia.

**Results:**

Twelve trypanorhynchan species were found, 10 in *R. jimbaranensis* and 8 in *R. penggali*, including the two new species *Prochristianella bicki* sp. nov. and *Pseudochristianella jimbaranensis* sp. nov.. While *Dollfusiella spinosa* Schaeffner & Beveridge, 2013 *Parachristianella indonesiensis* Palm, 2004, *Pa. monomegacantha* Kruse, 1959*, Proc. clarkeae* Beveridge, 1990*, Pseudochristianella jimbaranensis* sp. nov.*,* and *Trygonicola macropora* (Shipley & Hornell, 1906) occurred in both hosts, *Dollfusiella hemispinosa* Schaeffner & Beveridge, 2013*, Dollfusiella* sp., *Proc. bicki* sp. nov., and *Proemotobothrium linstowi* (Southwell, 1912) were only recorded from *R. jimbaranensis*, and *Dollfusiella michiae* (Southwell, 1929) and *Kotorelliella jonesi* Palm & Beveridge, 2002 were only found in *R. penggali*. Thirteen new host and eight new locality records are established.

**Conclusion:**

*Rhinobatos jimbaranensis* was infected with *Proe. linstowi* (Otobothrioidea), a species also infecting teleosts as intermediate hosts. While most recorded trypanorhynchs are transferred into *Rhinobatos* through the invertebrate hosts, this study suggests that *R. jimbaranensis*, similar to *R. penggali*, occasionally feeds on fish.

## Introduction

Indonesia, which has the world's highest level of marine biodiversity [1], is the largest archipelago and spans the Pacific and Indian Oceans and it lies on the Eurasian continental plate and borders the Australian plate [2]. With 221 species of sharks and rays its elasmobranch fauna is considered the richest in the world [3, 4]. As top predators, elasmobranchs play an important role in the dynamics of marine food webs and are essential for energy transfer within these systems [5–7]. They feed on a variety of organisms, including crustaceans and cephalopods, bony fish (Teleostei) and other elasmobranchs [8]. With reported landings of 105,000 and 118,000 tonnes in 2002 and 2003, respectively, Indonesia had the largest chondrichthyan fishery in the world [4]. Habitat loss, prey depletion due to overfishing, and direct fishing pressure have led to declines in many shark populations [9–11]. According to Dulvy et al. [12], about 40% of shark species are threatened with extinction.

Two endemic Indonesian rays are *Rhinobatos jimbaranensis* Last, White & Fahmi, 2006 and *R. penggali* Last, White & Fahmi, 2006. They inhabit the inner island shelves at depths of 1–60 m [13, 14]. *Rhinobatos jimbaranensis* so far has only been recorded in the eastern Indian Ocean off the southern coast of Bali, Indonesia [13, 14], with a single record from Perak Province, western peninsular Malaysia, not confirmed. It is listed by the IUCN red list of threatened species [15]. *Rhinobatos penggali* has been recorded from the southern coast of Java, Bali, and Lombok in the eastern Indian Ocean [13, 14]. Both species feed on small crustaceans, and *R. penggali* is suggested to also consume small bony fish [14].

A variety of parasitological studies on sharks and rays have been conducted in Indonesia. Fish parasites are commonly used as biological indicators of the host ecology, including migration, feeding behaviour, and zoogeographic distribution [16]. Elasmobranchs host a variety of parasites, especially cestodes, and the order Trypanorhyncha is of particular interest in this context [17]. The Trypanorhyncha community has been used for example, in combination with an analysis of the feeding ecology, to support conservation measures of elasmobranchs in Indonesian waters [18]. Trypanorhynchs are amongst the most species rich orders of marine cestodes and are distributed worldwide [19].

Only few *Rhinobatos* rays have been studied in Indonesia. Kleinertz et al. [18] examined five *R. penggali* from Penyu Bay in South Central Java and recorded the trypanorhynch *Parachristianella monomegacantha* Kruse, 1959 both as adults and as plerocerci. One of the better studied *Rhinobatos* species is *R. productus* (Ayres, 1854), where Campbell & Beveridge [20] revealed the presence of one trypanorhynch species in Santa Rosalía in the Gulf of California, Mexico, and Heinz and Dailey [21] reported 4 trypanorhynch species from the waters around California, USA. *Rhinobatos planiceps* (Garman, 1880) from Juan López Beach in Antofagasta, Chile [22] had 2 trypanoryhnch species, similar to *R. rhinobatos* (Linnaeus, 1758) [23]. The present study examined 12 *R. jimbaranensis* and 11 *R. penggali* from the southern coast of Bali, Indonesia. Observed differences in the trypanorhynch fauna between both species are discussed.

## Materials and Methods

Twenty-three Rhinobatidae were examined between November 2018 and September 2021. The rays were identified following Last et al. [13] as *Rhinobatos jimbaranensis* (12 specimens) and *R. penggali* (11 specimens). The specimens were purchased at the Pasar Ikan Tradisional Kedonganan market (8.757028 °S, 115.168389 °E) on the southern coast of Bali. The rays were transported on ice to the Laboratory of Aquatic Resource Management Study Program, Faculty of Marine Science and Fisheries, Udayana University, and examined following standardized methods [cf. 19, 24]. A gutwash methodology was used to collect all cestodes from the gastrointestinal tracts [25]. After removal from the spiral valve and stomach, the cestodes were fixed in 70% ethanol for morphological studies.

Before permanent mounting in Canada balsam, the trypanorhynchan were stained with Mayer–Schuberg’s acetic carmine [19, 26], and eugenol was used as the clearing agent. Photographs were taken with an Olympus DP74 digital camera attached to an Olympus BX53 DIC light microscope equipped with a drawing tube (camera lucida). CellSens 3.2 software (Olympus Soft Imaging Solutions GmbH) was used for measurements. Measurements in micrometres (range), followed by the mean and number (n) of measured characters in breckets. Descriptions follow Palm [19], with SL (scolex length), SW (scolex width), pbo (pars bothrialis), pv (pars vaginalis), pb (pars bulbosa), scolex ratio with pbo defined as 1 (pars bothrialis: pars vaginalis: pars bulbosa), BL (bulb length), BW (bulb width), BR (Bulb ratio) TL (tentacle length), TW (tentacle width), TS (tentacle sheat width), L (hook length), and B (base length).

Following Neitemeier‑Duventester et al. [27], a DMI6000 CS confocal laser scanning microscope (Argon/Krypton: 488 nm; He/Ne: 543 nm) equipped with a TCS SP5 II laser scanning unit (Leica Microsystems GmbH, Wetzlar, Germany) and a Leica Stellaris 8 confocal laser scanning microscope at the Institute of Biology, Zoology, Faculty of Mathematics and Natural Sciences, University of Rostock were used to create three‑dimensional images. Images were processed with IMARIS 9.9.1 software (Bitplane, Switzerland).

Selected specimens were examined by scanning electron microscopy (SEM) at the Scanning Electron Centre, Rostock University Medical Centre. For SEM preparation, specimens were transferred through a graded ethanol series to 100% ethanol and then to absolute acetone. CO₂ was used as the transitional fluid for critical‑point drying (Emitech K850, Quorum Technologies, East Sussex, UK). After mounting the specimens onto stubs using adhesive carbon tape (Plano, Wetzlar, Germany), they were coated with gold using a Safematic CCU‑010 sputter coater at 15 mA for 199 s in an argon atmosphere. Imaging was performed with a field‑emission SEM (Zeiss Merlin VP Compact) at a working distance of 4.1–4.9 mm. Images were acquired at 1000 × 1000 pixels with an integration time of 500 μs.

Parasitological and ecological parameters— intensity (I), mean intensity (mI), prevalence (P) and abundance (A)—were calculated according to Bush et al. [28]. Holotype and paratype material were deposited in the Zoological Museum Bogor, Indonesia (MZB), and additional paratype material in the Berlin Natural History Museum, Germany (ZMB).

## Results

Twelve trypanorhynch species were identified. In *Rhinobatos jimbaranensis* ten species were found: *Dollfusiella hemispinosa* Schaeffner & Beveridge, 2013*, D. spinosa* Schaeffner & Beveridge, 2013*, Dollfusiella* sp., *Parachristianella indonesiensis* Palm, 2004, *Pa. monomegacantha* Kruse, 1959*, Prochristianella bicki* sp. nov., *Proc. clarkeae* Beveridge, 1990, *Pseudochristianella jimbaranensis* sp. nov.*, Trygonicola macropora* (Shipley & Hornell, 1906) and *Proemotobothrium linstowi* (Southwell, 1912). In *R. penggali* eight species were identified: *Dollfusiella michiae* (Southwell, 1929)*, D. spinosa, Kotorelliella jonesi* Palm & Beveridge, 2002, *Pa. indonesiensis, Pa. monomegacantha, Proc. clarkeae, Ps. jimbaranensis* sp. nov. and *T. macropora*.

Among the identified Trypanorhyncha in *R. jimbaranensis, Ps. jimbaranensis* sp. nov. had the highest mean intensity (6.64), prevalence (83.33%) and abundance (6.08) (Table 1). In *R. penggali, Pa. monomegacantha* had the highest mean intensity (4.1) and abundance (3.42), and shared with *Ps. jimbaranensis* sp. nov. the highest prevalence (90.91%) (Table 1).

**Table 1:** Trypanorhyncha cestodes isolated from *Rhinobatos jimbaranensis* and *R. penggali* from Bali, Indonesia, with parasitological parameters

Parasites/Host	*Rhinobatos jimbaranensis* (n = 12)			*Rhinobatos penggali* (n = 11)		
	mI	P	A	mI	P	A
*Dollfusiella hemispinosa*	1	8.33%	0.08	–	–	–
*Dollfusiella michiae*	–	–	–	1	9.09%	0.09
*Dollfusiella spinosa*	1–2 (1.5)	16.67%	0.25	1	9.09%	0.18
*Dollfusiella* sp.	1	8.33%	0.08	–	–	–
*Kotorelliella jonesi*	–	–	–	2	9.09%	0.18
*Parachristianella indonesiensis*	1–6 (2.33)	50%	1.17	1	36.36%	0.36
*Parachristianella monomegacantha*	1–6 (2.13)	66.67%	1.42	1–9 (4.1)	90.91%	3.73
*Prochristianella bicki* sp. nov	3	8.33%	0.25	–	–	–
*Prochristianella clarkeae*	1–2 (1.25)	25%	0.42	1	9.09%	0.18
*Proemotobothrium linstowi*	1	8.33%	0.17	–	–	–
*Pseudochristianella jimbaranensis* sp. nov	1–22 (6.64)	83.33%	6.08	1–15 (3.89)	90.91%	3.18
*Trygonicola macropora*	1	16.67%	0.17	1–4 (2.5)	18.18%	0.45
Trypanorhyncha indet	1–21 (9.09)	91.67%	8.33	1–8 (3)	54.55%	1.64
Total Number of Trypanorhyncha	2–38 (18.42)	100%	18.42	2–26 (10)	100%	10.00

mI: mean intensity; P: prevalence; A: abundance

**Trypanorhyncha** Diesing, 1863

**Trypanobatoida** Olson, Caira, Jensen, Overstreet, Palm & Beveridge, 2010

**Eutetrarhynchoidea** Guiart, 1927

**Eutetrarhynchidae** Guiart, 1927

***Prochristanella*** Dollfus, 1946

**Fig. 1:**
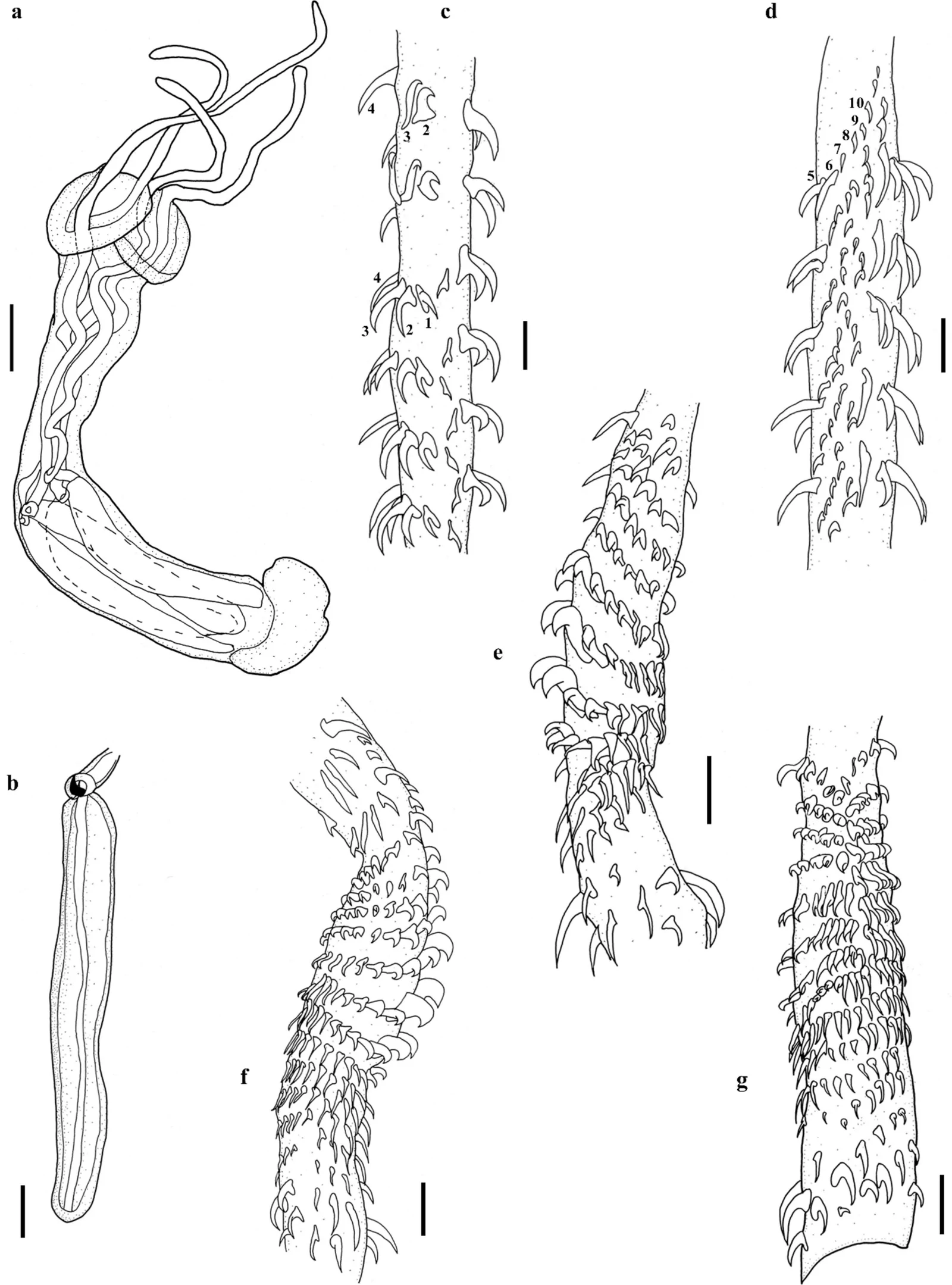
*Prochristianlla bicki* sp. nov. from spiral valve of *Rhinobatos jimbaranensis*. **a**: Scolex. **b**: Bulb with muscular ring. **c**: Metabasal armature, antibothrial surface, hooks 1(1’) disappear towards apical. **d**: Metabasal armature, bothrial surface. **e**: Basal armature, external surface, bothrial surface on left side. **f**: Basal armature, internal surface, bothrial surface on right side. **g**: Basal armature, antibothrial surface. Scale bars **a**: 200 µm; **b**: 100 µm; **c**–**g**: 20 µm

**Fig. 2:**
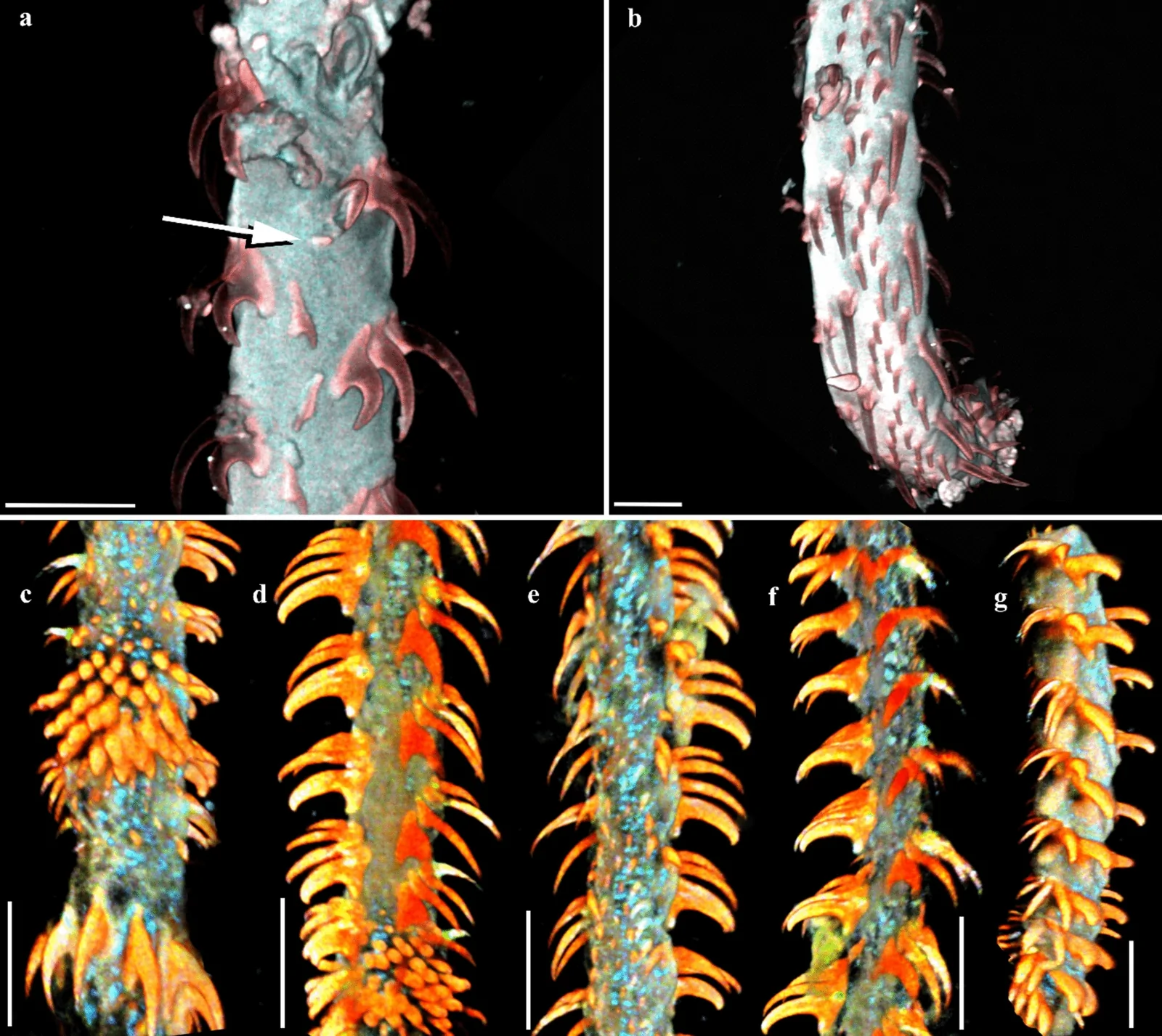
Confocal laser scanning microscope (CLSM) images. **a**, **b**: *Prochristianella bicki* sp. nov. from the spiral valve of *Rhinobatos jimbaranensis,* **a**: Metabasal armature, antibothrial surface, hooks 1(1’) disappear towards apical, in row 12 only a rudimentary hook visible (arrow). **b**: Metabasal armature, bothrial surface. **c**–**g**: *Pseudochristianella jimbaranensis* sp. nov. from the spiral valve of *Rhinobatos jimbaranensis,* c: Basal armature external surface. **d**: Basal to metabasal armature internal surface. **e**: Metabasal armature external surface. **f**: Metabasal armature internal surface. **g**: Metabasal armture bothrial surface. Scale bars: **a**–**c**: 20 μm; **d**: 30 μm; **e**: 20 μm; **f**: 30 μm; **g**: 20 μm

***Prochristianella bicki* sp. nov.** (Figs. 1 and 2)

**Taxonomic Summary**

Type host: *Rhinobatos jimbaranensis* Last, White & Fahmi, 2006.

Type locality: Kendonganan Fish Market, Bali, Indonesia (8.757028S, 115.168389E).

Site in host: Spiral valve.

Type material: Holotype (MZB, MZBCa 237); 2 paratypes (MZB, MZBCa 238 & ZMB, E. 7786).

ZooBank registration: urn:lsid:zoobank.org:act:2737E866-4A62-4CBF-8C43-2FF0582FBDF0.

Etymology: The specific name is in honour of Dr. Andreas Bick, former supervisor of the main author and his earlier work on the taxonomy of marine worms.

**Description**. Based on whole mounts of 3 specimens. Small worms, L = 1255–1883 (1629, n = 3), W max. = 199–270 (233, n = 3), microscopically visible prominent microtriches absent, strobila unknown. Scolex small (Figs. 1 a, 2 a), slender acraspedote, SL = 1200–1600 (1371), SW max. = 195–220 (207) at pb. 2 rounded, widely oval bothria, 200–290 (242) in diameter; bothrial pits absent. Pbo = 225–270 (242), pv = 580–915 (713), pb = 620–690 (650), scolex ratio 1:2.6–3.4:2.6–2.8 (1:2.92:2.7), ppb absent. Bulbs elongated (Fig. 1 b), BL = 613–687 (644, n = 9), BW = 49–62 (53,6, n = 9), BR = 10.7–14:1 (12.11:1, n = 9). Tentacles long, TL = 1219 (n = 1), TW metabasal = 24–34 (30, n = 6); basal swelling present, TW basal = 33–38 (34, n = 9). TS slightly sinuous. Prebulbar organs and gland cells present inside bulbs; retractor muscles originate at base of bulbs.

**Fig. 3:**
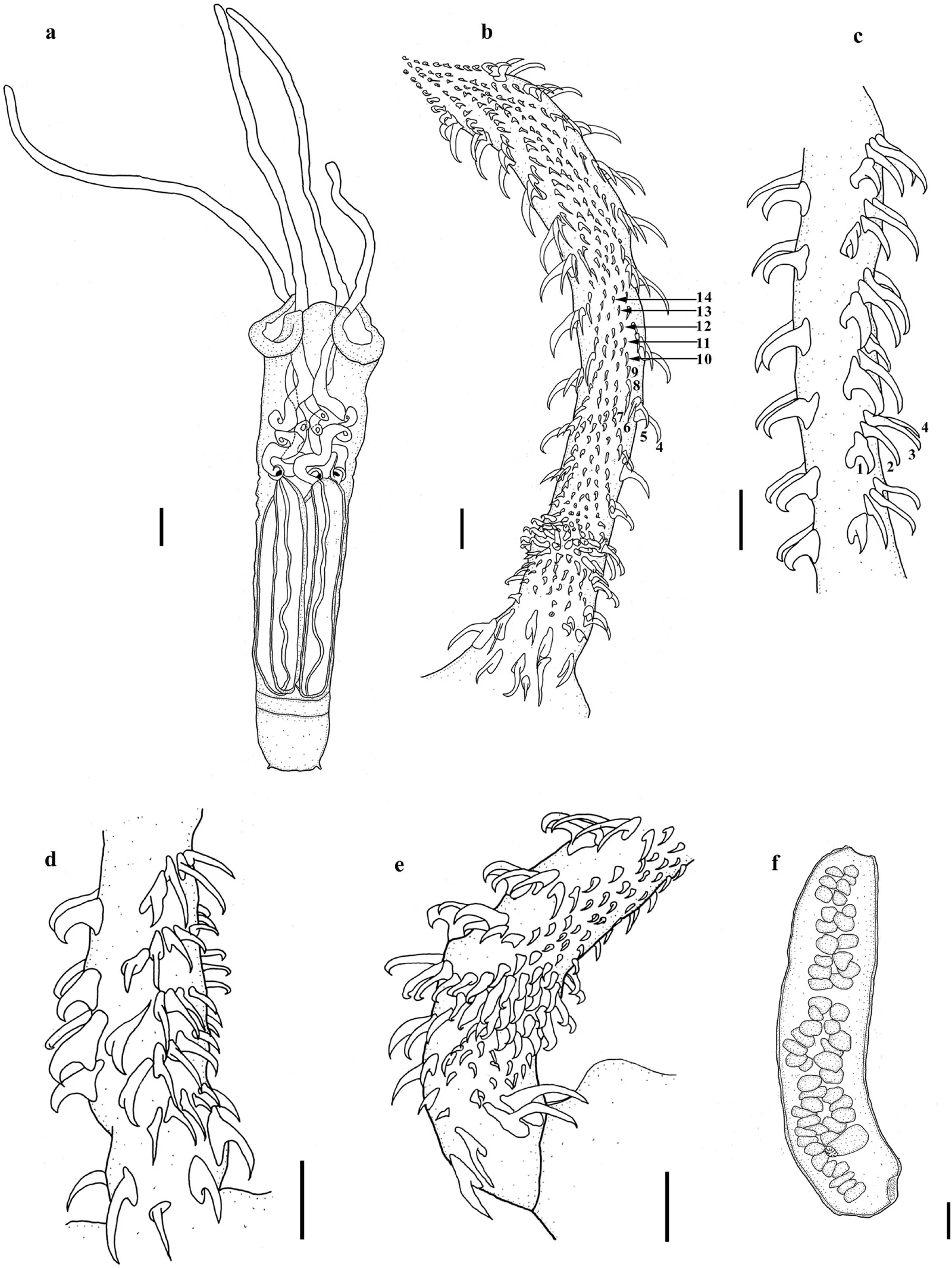
*Pseudochristianella jimbaranensis* sp. nov. from the spiral valve of *R. jimbaranensis.* **a**: Scolex. **b**: Basal to metabasal armature, external surface. **c**: Metabasal armature, internal surface. **d**: Basal armature, internal surface. **e**: Basal armature, external surface. **f**: Mature proglottid (paratype). Scale bars: **a**: 100 µm, **b**–**e**: 20 µm, **f**: 100 µm

Metabasal armature heteroacanthous typical, heteromorphous (Figs. 1 c-d, 2 b-c); hooks solid, arranged in ascending half spirals of 9–10 hooks. Rows begin on middle of antibothrial surface and terminate on middle of bothrial surface. Hooks 1 and 1’ uncinate, L = 6–9 (7.4, n = 8), B = 4–7 (5.4, n = 8), present only in first 11–12 rows of metabasal armature (Figs. 1 c, 3 b), in row 12 only a rudimentary hook visible (Fig. 2 b, arrow). Hooks 2(2’) larger, uncinate with anterior extension of the base, L = 10–14 (12.3, n = 10), B = 7–11 (8.5, n = 10). Hooks 3–6(3’-6’) larger, slender, gradually increasing in size, with much shorter anterior extension of the base; hooks 3(3’) L = 13–17 (14.8, n = 10), B = 5–7 (5.8, n = 10); hooks 4(4’) L = 17–21 (19, n = 10), B = 5–7 (6.3, n = 10); hooks 5(5’) L = 17–20 (18.8, n = 10), B = 4–7 (5.6, n = 10); hooks 6(6’) L = 15–20 (16.9, n = 10), B = 4–6 (5.1, n = 10); Hooks 7–10(7’-10’) small, uncinate, file not deviating towards anterior, hooks 7(7’) L = 5–7 (6.1, n = 10), B = 3–4 (3.6, n = 10); hooks 8(8’)-10(10’) smaller then hooks 7(7’), nearly of same size L = 4–6 (5.4, n = 10), B = 2–3 (2.9, n = 10).

Characteristic basal armature present (Figs. 1 e–g), hooks robust. First 2 rows of hooks enlarged, uncinate, L = 15–20 (17.5, n = 10), B = 7–12 (8.3, n = 10). Next 5–6 rows spiniform, L = 8–12 (10, n = 10), B = 3–4 (3.5, n = 10). Basal swelling with 6–7 rows of hooks, beginning on bothrial surface with enlarged robust uncinate hooks in first 3–4 rows, largest hook in first row L = 8–13 (11.2, n = 8), B = 7–13 (10.3, n = 8), largest hook in next row L = 10–12 (11.2, n = 8), B = 9–13 (11, n = 8), largest hook in next row L = 8–12 (9.4, n = 6), B = 8–11 (9.4, n = 6). Remaining 2–3 rows of hooks on basal swelling uncinate, decreasing in size towards antibothrial surface. Hooks become smaller uncinate with elongate base towards internal/external surfaces, followed by billhooks meeting on antibothrial surface. Metabasal armature originates from final billhooks on antibothrial surface, with hooks 1(1´) small uncinate with distinct base.

**Remark:**

Three specimens of *Prochristianlla bicki* sp. nov. were found in the spiral valve of *R. jimbaranensis*.

*Prochristianella bicki* sp. nov. is assigned to the genus *Prochristianella* following Beveridge et al. [23], due to the heteroacanthous typical armature, with widely separated hooks 1 and 1’ on the antibothrial surface, and the metabasal principal rows terminating in inverted V-form on the bothrial surface (Figs. 1 d, 2 c).The species is also shares the most important feature of the genus, that the metabasal hooks increase in size on the antibothrial surface and then decrease towards the bothrial surface (hooks 1(1’) L = 8; hooks 5(5’) L = 18.8; hooks 8–10(8’-10’) L = 5.4) [23, 29].

The new species differs from other *Prochristianella* species in having hooks 1(1’) disappear beyond metabasal hook row 11 and hooks arranged in ascending half spirals of 9–10 hooks. A characteristic basal armature is present with 4 rows of enlarged billhook shaped hooks on the bothrial surface of the basal swelling. The absence of microscopically visible prominent microtriches distinguishes *Proc. bicki* sp. nov. from *Proc. hispida* (Linton, 1890), *Proc. clarkeae* Beveridge, 1990, *Proc. thalassia* (Kovacs & Schmidt, 1980) and *Proc. cairae* (Schaeffner & Beveridge, 2012) (Palm, 2004; Schaeffner & Beveridge, 2012). *Prochristianella scholzi* (Schaeffner & Beveridge, 2012) has hollow hooks [29] compared with the solid hooks of *Proc. bicki* sp. nov. The morphologically most similar species is *Proc. mooreae* Beveridge, 1990, also with enlarged billhooks on the basal swelling and having metabasal hooks 1(1’) and 2(2’) disappearing apically. Despite these similarities, *Proc. bicki* sp. nov. can be clearly distinguished from *Proc. mooreae* by having 9–10 solid hooks arranged in ascending half spirals compared with 10–12 hollow hooks [30]. Besides this, hooks 1(1’) are uncinate compared with spiniform in *Proc. mooreae* and hooks 2(2’) do not disappear apically as in *Proc. mooreae*.

***Pseudochristianella*** Campbell & Beveridge, 1990

**Fig. 4:**
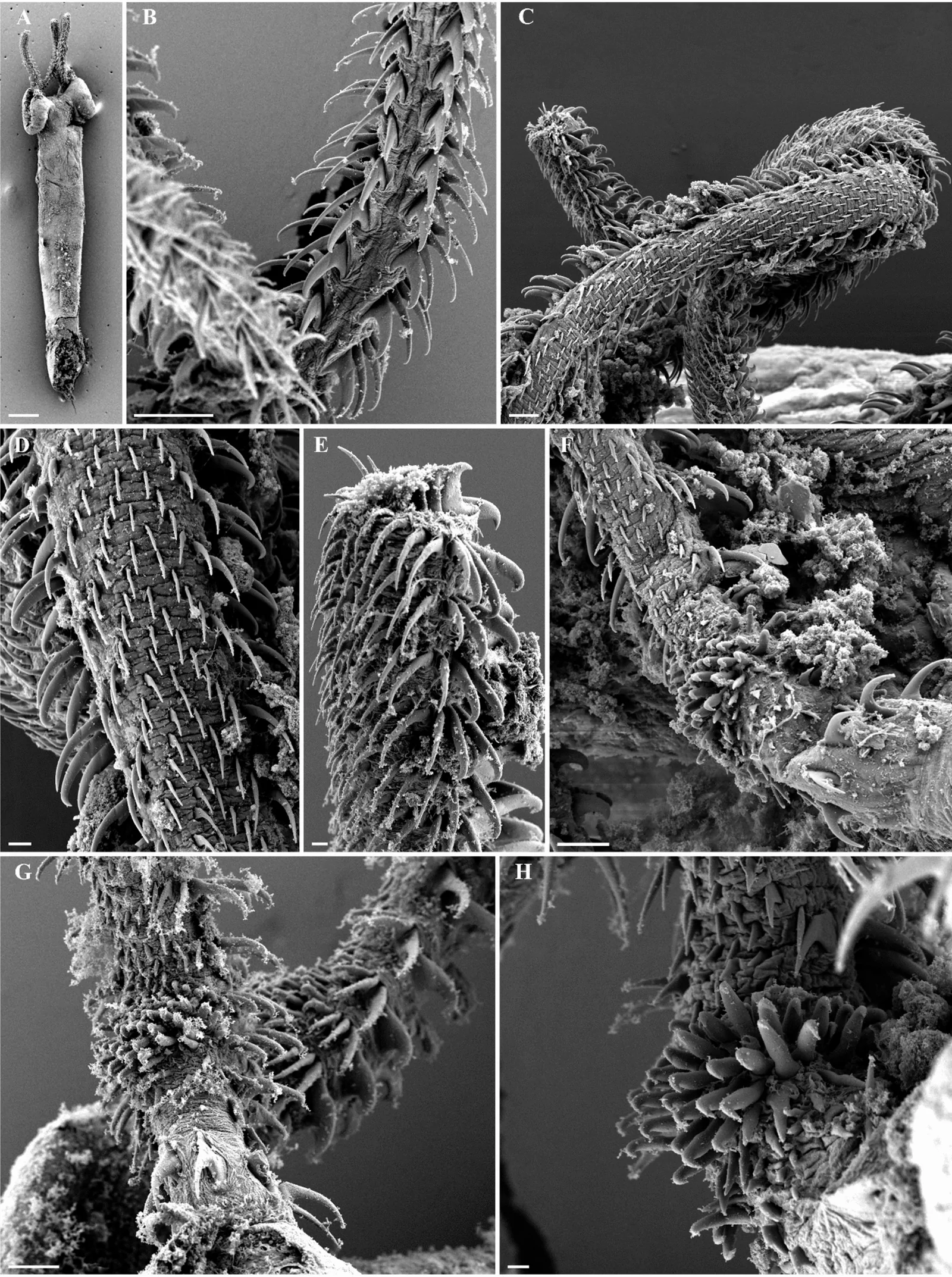
Scanning Electron microscopy (SEM) images of *Pseudochristianella jimbaranensis* sp. nov. from the spiral valve of *Rhinobatos jimbaranensis.* **a**: Scolex. **b**: Metabasal armature, internal surface. **c**: Metabasal armature, external surface. **d**: Metabasal armature, external surface. **e**: Metabasal armature, bothrial surface. **f**: Basal armature, internal surface. **g**: Basal armature, internal surface. **h**: Basal armature, internal surface. Scale bars: **a**: 100 µm; **b**:20 µm; **c**: 10 µm; **d**: 3 µm; **e**: 2 µm; **f**: 10 µm: **g**: 10 µm: **h**: 2 µm

***Pseudochristianella jimbaranensis* sp. nov.** (Figs. 2, 3 and 4)

**Taxonomic Summary**

Type host: *Rhinobatos jimbaranensis* Last, White & Fahmi, 2006

Additional host: *Rhinobatos penggali* Last, White & Fahmi, 2006

Type locality: Kedonganan Fish Market, Bali, Indonesia (8.757028S, 115.168389E)

Site in host: Spiral valve

Type material: Holotype (MZB, MZBCa 239), XXX paratypes (MZB, MZBCa 240, 241 & ZMB, E. 7783-7785)

ZooBank registration: urn:lsid:zoobank.org:act:5C324B5F-80F6-471A-911D-2D43329F6530

Etymology: The specific name refers to the locality and zoogeographic distribution of the parasite, also referring to the name of the type host, *Rhinobatos jimbaranensis*.

**Description.** Based on 14 whole mounts and 6 specimens prepared for SEM. Small worms L = 714–4249 (1221, n = 11), W = 135–349 (200, n = 11), with 4–6 proglottids, acraspedote. Scolex small, slender acraspedote (Figs. 2 d, 3 a, 4 a), SL = 714–1078 (878.3), SW max. = 118–242 (17.7) at pbo. Two rounded, oval bothria, 138–192 (167.5, n = 13) in diameter; bothrial pits absent. Pbo = 135–201 (164.6), pv = 331–582 (439.5), pb = 299–587 (441.4), scolex ratio 1:2.4–3.4:1.9–3.2 (1:2.87:2.59), ppb absent. Bulbs elongated, BL = 262–502 (388.6, n = 12), BW = 35–64 (45, n = 12), BR = 5.7–12.6:1 (8.7:1). Tentacles long TL = 599–893 (698.9, n = 3), TW = 18–30 (24.4, n = 12) metabasal; small basal swelling present, TW = 19–30 (25.6, n = 12). TS slightly sinuous. Prebulbar organs and gland cells present inside bulbs; retractor muscles originate at base of bulbs.

Metabasal armature heteroacanthous typical, heteromorphous (Figs. 2 e–g, 3 b-c, 4 b-f); hooks solid, arranged in ascending half spirals of 15 hooks above basal armature, reducing to 13 distally, rows overlap and alternate on external surface of tentacle, appearing as band of hooks 4 rows wide. Rows begin on middle of internal surface and terminate on middle of external surface, with distinct space between hooks 1 and 1’. Hooks 1 and 1’ uncinate, L = 15–18 (16.9, n = 10), B = 19–13 (10.7, n = 10). Hooks 2–3(2’-3’), slenderer uncinate to falciform, gradually decreasing in size. Hooks 2(2’) largest in row, L = 15–21 (18.5, n = 10), B = 4–7 (5.7, n = 10); hooks 3(3’) L = 15–20 (17.7, n = 10), B = 4–7 (5.2, n = 10); hooks 4(4’) slender uncinate, L = 11–16 (13, n = 10), B = 3–5 (3.9, n = 10). Hooks 5(5’)–6(6’) slender uncinate, hooks 5(5’) L = 7–12 (8.8, n = 10), B = 2–4 (2.8, n = 10). Row with hooks 6–15(6’-15’) change direction towards apical, hooks 6(6’) L = 5–8 (6.1, n = 10), B = 1.6–2.2 (1.9, n = 10), 7–15(7’-15’). Hooks 7–15(7’-15’) roughly of same size, L = 3–6 (3.7, n = 35), B = 1.5–2.4 (1.8, n = 35).

Characteristic basal armature present (Figs. 2 e–f, 3 b, d-f, 4 f–h). First 2 rows of enlarged slender uncinate hooks, L = 18–23 (20.7, n = 10), B = 11–15 (12.7, n = 10), followed by 4 rows of small spiniform hooks before basal swelling on external surface. Internal hooks of rows 2–3 robust, enlarged uncinate, L = 11–17 (13.6, n = 10), becoming slender uncinate bothrial/antibothrial, forming 4 rows of billhooks on external surface of basal swelling, total number of billhooks appr. 35–36.

First proglottid short, directly behind pas proliferans, L = 23–121 (65.8, n = 5); W = 126–168 (142.2, n = 5), wider than long, followed by immature proglottid without distinct genital organs, longer than wide, L = 140–468 (279.8, n = 3); W = 139–215 (171.9, n = 3). Third proglottid mature (Fig. 3 e), L = 478–952 (665.6, n = 3); W = 206–260 (236, n = 3), genital pore not seen. Testes aligned in two columns; appr. 48–52 testes, L = 48–65 (56.6, n = 10), W = 34–54 (42.6, n = 10). Cirrus sac pear-shaped with thick wall, L = 119; W = 85; cirrus unarmed; internal and external seminal vesicles not seen. Osmoregulatory canals: ventral canal straight. Proglottids 4–6 gravid, L = 445–1201 (811.8, n = 6); W = 342–514 (417.1, n = 6).

**Remark:**

Seventy-three specimens of *Pseudochristianella jimbaranensis* sp. nov. were found in the spiral valve of *Rhinobatos jimbaranensis* and 35 specimens in the spiral valve of *R. penggali*. *Pseudochristianella jimbaranensis* sp. nov. is assigned to the genus *Pseudochristianella* following Campbell & Beveridge [20] due to its basal swelling with a distinctive basal armature with symmetrically-arranged billhooks at the termination of one or more hook rows. *Pseudochristianella jimbaranensis* sp. nov. can be distinguished from the 3 already described species by having 2 rows of enlarged slender uncinate hooks at the base, followed by 4 rows of small spiniform hooks before basal swelling on external surface; internal hooks of rows 2–3 are robust, enlarged uncinate, becoming slender uncinate bothrial/antibothrial, forming 4 rows of billhooks on the external surface of the basal swelling, with a total number of billhooks of appr. 35–36. *Pseudochristianella elegantissima* has a similar metabasal armature with 13 hooks per principal row, as in 13–15 in *Ps. jimbaranensis* sp. nov. However, the basal armature can be clearly distinguished. *Pseudochristianella elegantissima* has 2–3 enlarged billhooks only in row 6 [20]. The metabasal armature of *Pseudochristianella nudiscula* is distinguishable from *Ps. jimbaranensis* sp. nov. by having only 10 hooks per principal row, and the basal armature is clearly distinguishable as well by having billhooks arranged on the external surface as alternating pairs with small spiniform hooks interspersed [20]. The new species is most similar to *Ps. southwelli*. However, the metabasal armature differs with only 11 hooks per principal row, and the number of about 20 billhooks is much lower than 35 billhooks in *Ps. jimbaranensis* sp. nov. [19]. The number of testes with about 80 in *Ps. southwelli* is also higher compared with 48–52 in *Ps. jimbaranensis* sp. nov. [31]*.* Because the proglottids are over stained, many important characters can be recognised poorly or not at all.

**Other parasite records**

**Trypanorhyncha** Diesing, 1863

**Trypanobatoida** Olson, Caira, Jensen, Overstreet, Palm & Beveridge, 2010

**Eutetrarhynchoidea** Guiart, 1927

**Eutetrarhynchidae** Guiart, 1927

***Dollfusiella*** Campbell & Beveridge, 1994

***Dollfusiella hemispinosa*** **Schaeffner & Beveridge, 2013**

Morphological vouchers: ZMB, E. 7787. 

One specimen of *Dollfusiella hemispinosa* was found in the spiral valve of *Rhinobatos jimbaranensis*. The morphology and morphometry matched the description provided by Schaeffner & Beveridge [32]. Most characteristic were the enlarged microtriches extending on the scolex surface and the characteristic basal armature with the first row of enlarged uncinate hooks and characteristic billhooks on the antibothrial surface of the basal swelling. The heteroacanthous, homeomorphous metabasal armature consists of 10 falcate, hollow hooks. The type host of *D. hemispinosa* is *Maculabatis pastinacoides* (Bleeker, 1852), and additional host records are *Pateobatis uarnacoides* (Bleeker, 1852) (as *Himantura uarnacoides* in Schaeffner & Beveridge [32]) and *H. uarnak* 3 (sensu Naylor et al., 2012) from Malaysia (South China Sea off Sematan, Sarawak; South China Sea off Mukah, Sarawak and Sulu Sea off Sandakan, Sabah [32]. This report of *D. hemispinosa* from *R. jimbaranensis* from the Kedonganan fish market in Bali, Indonesia, represents a new host and locality record.

***Dollfusiella michiae*** **(Southwell, 1929)**

Morphological vouchers: ZMB, E. 7788. 

One specimen of *Dollfusiella michiae* was found in the spiral valve of *Rhinobatos penggali*. The morphology and morphometry matched the description provided by Beveridge [30]. *Dollfusiella michiae* was recorded from *Neotrygon kuhlii* (Müller & Henle, 1841) *Pastinachus sephen* (Forsskål, 1775) from Sri Lanka [19] and from *Urogymnus lobistoma* (Manjaji-Matsumoto & Last, 2006), *Himantura undulata* (Bleeker, 1852) and *Pastinachus ater* (Macleay, 1883) from Borneo [33]. Neitemeier-Duventester et al. [27] illustrated *D. michiae* from *Neotrygon caeruleopunctata* Last, White & Serét, 2016 also from the Kedonganan fish market in Bali, Indonesia. *Rhinobatos penggali* represents a new host record for *D. michiae.*

***Dollfusiella hemispinosa*** **Schaeffner & Beveridge, 2013**

Morphological vouchers: ZMB, E. 7789,7790. 

Three specimens of *Dollfusiella spinosa* were found in the spiral valve of *Rhinobatos jimbaranensis* and 2 specimens in the spiral valve of *R. penggali.* The morphology and morphometry matched the description provided by Schaeffner & Beveridge [32]. Most characteristic were the enlarged microtriches on the scolex surface and the characteristic metabasal armature first 4 rows of enlarged uncinate hooks and billhooks on the bothrial surface on the basal swelling. The heteroacanthous, homeomorphous metabasal armature consists of 8 falcate, hollow hooks. The type host of *D. spinosa* is *Pastinachus solocirostris* Last, Manjaji & Yearsley, 2005 from the South China Sea off Mukah, Sarawak, Malaysia, additional hosts are *Taeniura lymma* 1 (sensu Naylor et al., 2012), *Neotrygon orientale* Last, White & Serét, 2016 [in 32] as *N. kuhlii* 2 (sensu Naylor et al., 2012), *Glaucostegus* cf. *typus* (sensu Naylor et al., 2012) and additional localities from Malaysia are the South China Sea off Sematan, Sarawak, Celebes Sea off Pulau Mabul, Sabah, Kinabatangan River near Celebes Sea, Sabah and Sulu Sea off Kampung Tetabuan, Sabah [32]. This report of *D. spinosa* represents new host and locality records for *R. penggali* and *R. jimbaranensis* from the Kedonganan fish market in Bali, Indonesia.

***Dollfusiella sp***

Morphological vouchers: MZBCa 242.

In addition to *Dollfusiella hemispinosa* and *D. spinosa*, a third *Dollfusiella* species was detected in *Rhinobatos jimbaranensis*. A single specimen was found in the spiral valve which could clearly distinguished from *D. hemispinosa*, *D. michiae* and *D. spinosa*. The specimen is damaged and the complete scolex could not be analysed. SL = 508, pbo = 85, pv = 262, pb not complet. 2 oval bothria, with free posterior margins. Scolex lacks enlarged microtriches. First 3–4 rows of enlarged hooks, basal swelling present.

***Parachristianella*** Dollfus, 1946.

***Parachristianella indonesiensis*** **Palm, 2004**

Morphological vouchers: ZMB, E. 7791. 

Fourteen specimens of *Parachristianella indonesiensis* were found in the spiral valve of *Rhinobatos jimbaranensis* and 4 specimens in the spiral valve of *R. penggali*. The morphology and morphometry matched the description provided by Palm [19]. Most characteristic were 2 enlarged billhooks on the basal armature. Palm [19] reported *Pa. indonesiensis* from *Himantura* sp., *Pastinachus sephen* (Forsskål, 1775) and *T. lymna* from Pelabuhan Ratu, Java, Indonesia. The report of *Pa. indonesiensis* from *Past. sephen* most likely represents a host misidentification. *Pastinachus* was long regarded as a monotypic genus [34]. According to current knowledge [14], *Past. sephen* does not occur in Indonesia. Schaeffner & Beveridge [33] reported *Pa. indonesiensis* from *Past. solocirostris* in Borneo; *Past. solocirostris* also occurs on Java and, according to Last et al. [14], is the only *Pastinachus* species present there. Therefore, the record reported by Palm [19] most likely also refers to *Past. solocirostris*. This report of *Pa. indonesiensis* from *R. jimbaranensis* and *R. penggali* from the Kedonganan fish market in Bali, Indonesia, represents new hosts and locality records.

***Parachristianella monomegacantha*** **Kruse, 1959**

Morphological vouchers: ZMB, E. 7792, 7793. 

Seventeen specimens of *Parachristianella monomegacantha* were found in the spiral valve of *Rhinobatos jimbaranensis* and 41 specimens in the spiral valve of *R. penggali*. The morphology and morphometry matched the description provided by Palm [19]. Most characteristic were the first two rows of large uncinate, strongly recurved hooks and the metabasal armature directly beginning after the first two basal hook rows with hooks 1(1’) large uncinate, strongly recurved with large base. *Parachristianella monomegacantha* is worldwide distributed [19] and had already been reported from other *Rhinobatos* species and regions [20, 22, 23]. Schaeffner & Beveridge [33] reported *Pa. monomegacantha* in serval different hosts from Borneo. Heinz & Daily [21] and Campbell & Carvajal [35] reported it from *R. productus*. According to Beveridge et al. [23], *Pa. monomegacantha* was observed in *R. rhionobatos* in Zarzis, Tunisia. Kleinertz et al. [18] reported *Pa. monomegacantha* from *R. penggali* from Penyu Bay, Java, Indonesia, making it the most common trypanorhynch for this ray genus. Theisen [36] reported *Pa. monomegacantha* from *Plectorhinchus gibbosus* (Lacépède, 1802) from Bali. This report of *Pa. monomegacantha* represents a new host record for *R. jimbaranensis*.

***Prochristianella*** Dollfus, 1946

***Prochristianella clarkeae*** **Beveridge, 1990**

Morphological vouchers: ZMB, E. 7794, 7795

Five specimens of *Prochristianella clarkeae* were found in the spiral valve of *Rhinobatos jimbaranensis* and 2 specimens in the spiral valve of *R. penggali.* The morphology and morphometry matched the description provided by Beveridge [30]. Most characteristic were the 3 rows of enlarged triangular shaped hooks on the basal swelling. *Himantura uarnak* (Gmelin, 1789) from Fog Bay, Australia was given as type host. *Prochristianella clarkeae* has a Indo-west Pacific and Australian distribution [33], there are serval host records from Borneo [33], as well as from Australia [19]. Additionaly, Palm [19] reported *Proc. clarkeae* from *Dasytbaus* sp. from pearl Banks, Sri Lanka. *Prochristianella clarkeae* was earlier found in *Rhinobatos* sp. from another region, off Weipa, Queensland, Australia [29]. This report of *Proc. clarkeae* from *R. jimbaranensis* and *R. penggali* from the Kedonganan fish market in Bali, Indonesia, represents new host and locality records.

**Mixodigmatidae** Dailey & Vogelbein, 1982.

***Trygonicola*** Beveridge & Campbell, 1998.

***Trygonicola macropora*** **(Shipley & Hornell, 1906)**

Morphological vouchers: ZMB, E. 7796, 7797 .

Two specimens of *Trygonicola macropora* were found in the spiral valve of *Rhinobatos. jimbaranensis* and 5 specimens in the spiral valve of *R. penggali*. The morphology and morphometry matched the description provided by Beveridge & Campbell [37]. Palm [19] reported *T. macropora* from *Aetobatus narinari* (Euphrasen, 1790) from Kuala Lumpur, Malaysia and several hosts from Sri Lanka. Schaeffner & Beveridge [33] reported *T. macropora* from several hosts from Borneo. Neitemeier-Duventester et al. [27] illustrated *T. macropora* from *N. caeruleopunctata* also from the Kedonganan fish market in Bali, Indonesia. This report of *T. macropora* represents new host records for *R. penggali* and *R. jimbaranensis.*

**Tentacularioidea** Poche, 1926.

**Tentaculariidae** Poche, 1926.

***Kotorelliella*** Palm & Beveridge, 2002.

***Kotorelliella jonesi*** **Palm & Beveridge, 2002**

Morphological vouchers: ZMB, E. 7798.

Two specimens of *Kotorelliella jonesi* were isolated from the spiral valve of *Rhinobatos penggali.* The morphology and morphometry matched the description provided by Palm & Beveridge [38]. Most characteristic were the elongated scolex shape with ellipsoid bothria not overlapping the pb and the homeoacanthous metabasal armature, and heterocanthous atypical basal armature. The type host of *K. jonesi* is *Taeniura lymma* (Forsskal, 1775) from the Heron Island, Queensland, Australia. This report of *K. jonesi* from *R. penggali* from the Kedonganan fish market in Bali, Indonesia, represents a new host and locality record.

**Trypanoselachoida** Olson, Caira, Jensen, Overstreet, Palm and Beveridge, 2010.

**Otobothrioidea** Dollfus, 1942.

**Otobothriidae** Dollfus, 1942.

***Proemotobothrium*** Beveridge & Campbell, 2001.

***Proemotobothrium linstowi*** **(Southwell, 1912)**

Morphological vouchers: ZMB, E. 7799.

Two specimens of *Proemotobothrium linstowi* were found in the spiral valve of *Rhinobatos jimbaranensis*. The morphology and morphometry matched the description provided by Beveridge & Campbell [39]. Most characteristic were the large bothrial pits, a characteristic basal armature with 3 ascending rows of hooks, and the uncinate hooks with blunt tips on the bothrial and antibothrial surface. Metabasal 2 tandem hooks were seen on the external surface at the end of each principal row. The type host is *Rhynchobatus djiddensis* (Forsskål, 1775) from Sri Lanka [40], additional locality records from Australia were given by Beveridge & Campbell [39]: Broome, Cape van Diemen, Fog Bay, Mackay, Flat top Island (Queensland) and Heron Island. Also, these authors recorded the species for the shark *Carcharhinus* sp. from Sri Lanka Negapatnam. *Proemotobothrium linstowi* was reported from *Urogymnus polylepis* (Bleeker, 1852) and *Rhynchobatus cf. laevis* (Bloch & Schneider, 1801) from Borneo [33]. This report of *Proe. linstowi* from *R. jimbaranensis* from the Kedonganan fish market in Bali, Indonesia, represents a new host and locality record.

## Discussion

Not much is known about the Trypanorhyncha fauna from *Rhinobatos* elasmobranchs. The best studied species with the highest number of recorded trypanorhynch species is *R. productus* with 5 trypanorhynch species so far. Heinz & Dailey [21] reported *Lacistorhynchus dollfusi* Beveridge & Sakanari, 1987, *Dollfusiella schmidti* (Heinz & Dailey, 1974), *Parachristianella monomegacantha*, and *Prochristianella fragilis* (Heinz & Dailey 1974) from California, USA, and Campbell & Beveridge [20] added *Pseudochristianella nudiscula* from Santa Rosalia and Bahía de Los Angeles, Gulf of California, Mexico. Two species have been recorded from *R. planiceps* and *R. rhinobatos* respectively. *Parachristianella monomegacantha* and *Proc. heteracantha* Dailey & Carvajal, 1976 were reported from *R. planiceps* from Juan López Beach in Antofagasta, Chile [22], and *D. elongata* Beveridge, Neifar & Euzel, 2004 and *Pa. monomegacantha* from *R. rhinobatos* from Zarzis, Tunisia [23]. *Parachristianella baverstocki* Beveridge, 1990, *Proc. clarkeae* and *Pterobothrium acanthotrunctatum* Escalante & Carvajal, 1984 were reported from *Rhinobatos* cf. *laevis* [19, 29]. It is evident, that these rays are mainly parasitised by the trypanorhynch genera *Dollfusiella*, *Prochristianella*, *Parachristianella* and *Pseudochristianella*, with a range of additional genera belonging to the Eutetrarhynchoidea and few Lacistorhynchoidea that also infect these elasmobranchs.

The only trypanorhynch reported so far from *R. penggali* was *Pa. monomegacantha* from Penyu Bay, Java, Indonesia [18], while *R. jimbaranensis* had not been studied before. Within the present study, 10 trypanorhynch species (of a total of 12) were found in *R. jimbaranensis* and 8 in *R. penggali*, with *D. spinosa*, *Pa. indonesiensis*, *Pa. monomegacantha*, *Proc. clarkeae*, *Ps. jimbaranensis* sp. nov., and *Trygonicola macropora* infecting both hosts. The most common parasite in both ray species was *Ps. jimbaranensis* sp. nov., indicating that this species must be a widespread parasite of *Rhinobatos* in Bali. Also the other 5 trypanorhynch species infecting both *Rhinobatos* have been already reported elsewhere in Indonesia or in the Indo-Pacific. Still, it is evident that the trypanorhynch diversity in *Rhinobatos* from Balinese waters is much (double) higher than reported so far from any other region.

With 4 of the 12 recorded species, the genus *Dollfusiella* was well represented within this sampling. The prevalences and abundances of *Dollfusiella*, however, were low in both species of *Rhinobatos* (Table 1). This suggests that they are non-core species. By contrast, species of *Parachristianella* and *Pseudochristianella* exhibited much higher prevalences in both species of host (Table 1) and appear to be core species in Balinese *Rhinobatos*. The predominance of eutetrarhynchoids in the parasite fauna of both ray species is consistent with their widespread use of crustaceans as intermediate hosts, including *Pa. monomegacantha*. According to Last et al. [14], the diet of *R. jimbaranensis* consists primarily of crustaceans while that of *R. penggali* includes both crustaceans and fish. The analyses of the stomach contents of *R. penggali* confirmed this assumption [18]. However, the Tentaculariidae (*Kotorelliella*) and Otobothriidae (*Proemotobothrium*) typically have teleost intermediate hosts (Palm, 2004). While the life cycle of *Kotorelliella* so far is unknown [19], the plerocercus of *Proe. linstowi* has been reported from teleosts [39]. The latter was recorded only from *R. jimbaranensis* and not *R. penggali*, suggesting that the former ray species, similar to its congener, also includes fish in its diet.

Even though there are some studies on the Trypanorhyncha cestode fauna from Indonesian elasmobranchs, the knowledge on the fauna from Bali is still scarce. Kleinertz et al. [18] reported 4 trypanorhynch species (only two identified to species level) (*Dollfusiella* sp., *Kotorella pronosoma* (Stossich, 1901), *Pa. baverstocki* and *Prochristianella* sp.) from *Maculabatis gerradi* (Gray, 1851) also from Kedonganan fish market, and Neitemeier-Duventester et al. [27] found *D. michiae* and *T. macropora* from *Neotrygon caeruleopunctata* also from Jimbaran. Other records originate from the characteristic trypanorhynch fauna found in *Mobula* manta rays (Palm et al. [41] and Suryanintyas et al. [42, 43]). While the herewith description of 2 new species suggests a high diversity of trypanorhynchs in Bali, the very few published surveys do not allow regional comparisons (e.g. Borneo, see Schäffner & Beveridge [33]). Further studies are needed in order to explore the full species diversity of trypanorhynchs in Balinese waters.

## Data Availability

No datasets were generated or analysed during the current study.
